# A guide to sequence your favorite plant genomes

**DOI:** 10.1002/aps3.1030

**Published:** 2018-03-30

**Authors:** Fay‐Wei Li, Alex Harkess

**Affiliations:** ^1^ Boyce Thompson Institute Ithaca New York 14853 USA; ^2^ Plant Biology Section Cornell University Ithaca New York 14853 USA; ^3^ Donald Danforth Plant Science Center St. Louis Missouri 63132 USA

**Keywords:** genome assembly, Hi‐C, Illumina, Nanopore, optical mapping, PacBio

## Abstract

With the rapid development of sequencing technology and the plummeting cost, assembling whole genomes from non‐model plants will soon become routine for plant systematists and evolutionary biologists. Here we summarize and compare several of the latest genome sequencing and assembly approaches, offering a practical guide on how to approach a genome project. We also highlight certain precautions that need to be taken before investing time and money into a genome project.

Sequencing and assembling a complete plant genome has been seen as a daunting task. Indeed, the first plant genome of *Arabidopsis thaliana* (L.) Heynh. took 10 years to finish and cost approximately US$100 million (Goff et al., 2014). The current generation of DNA sequencing technologies, however, is making genome sequencing a reality even for small labs without generous funding sources. For instance, a high‐quality *A. thaliana* genome can now be sequenced with a USB device on a regular laptop at a cost of under US$1000, with de novo assembly complete within a week (Michael et al., 2017). This momentous leap brings exciting opportunities to the botanical community. Whole genomes, paired with resequencing, can provide thousands of nuclear markers for phylogenetic and population‐level studies, enabling genome‐wide investigations into fundamental evolutionary and ecological questions. In addition, generating a pan‐genome—capturing the genomic diversity of ecotypes, geographical isolates, and related species (Golicz et al., 2016)—will make comparative approaches and association studies possible to identify the genetic components of certain traits and adaptations. The possibilities run the gamut from systematics, ecology and evolution, to molecular genetics.

Despite the dramatic drop in sequencing cost and the rise in throughput and read length, care still needs to be taken when planning a genome project in order to maximize assembly quality versus cost. In this review, we first illustrate the necessary measures that need to be considered before sequencing, describe several current sequencing approaches and strategies, and provide an overview of genome assembly techniques. Note that the cost estimates mentioned in this review were based on our quote inquiries from several service providers and were made between July to November 2017. These numbers are likely to decrease through time.

## BEFORE SEQUENCING

Not all plants are equally sequenceable. Genome size, repeat structure and age, and heterozygosity are the three main factors that determine the feasibility of the project. In order to strategize the sequencing approach, certain groundwork is necessary.

### Genome size and complexity

Plant genome sizes vary dramatically, ranging from 0.063 to 148.8 Gbp (Greilhuber et al., 2006; Hidalgo et al., 2017), and the sequencing cost increases as the genome size increases. Indeed, only a few genomes larger than 10 Gbp have been assembled, such as wheat (Zimin et al., 2017a, 2017b), *Ginkgo* L. (Guan et al., 2016), *Picea* A. Dietr. (Birol et al., 2013; Nystedt et al., 2013), and *Pinus* L. (Zimin et al., 2017c). In addition, assembly of allo‐ or autopolyploid genomes is complicated by the presence of additional haplotypes. Therefore, identifying a haploid or diploid individual with a relatively small genome in your clade of interest is critical; this can not only save significant amounts of money, but also simplify downstream bioinformatics analyses. However, if such individuals are not available or if polyploids are actually the targets, one should consider long‐read sequencing coupled with Hi‐C, optical mapping, or 10× Genomics (10× Genomics Inc., Pleasanton, California, USA) (see Discussion).

**Figure 1:**
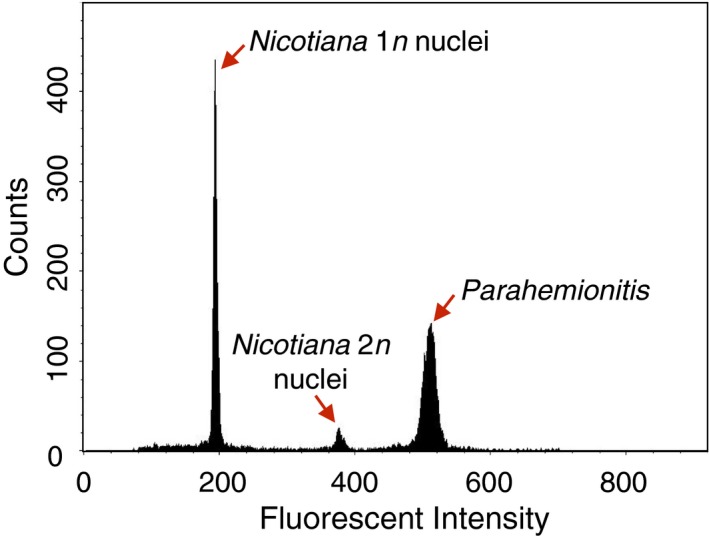
An example flow cytometry result, using *Nicotiana* as the standard to infer the genome size of the fern *Parahemionitis cordata*.

Flow cytometry (Fig. 1) is a common and accurate way to determine genome size, but it requires fresh material and buffer optimization (see Dolezel and Bartos, 2005). External groups such as the Benaroya Research Institute (Seattle, Washington, USA) have significant experience with fast, low‐cost (US$15) plant genome sizing. The Royal Botanic Gardens, Kew Plant DNA C‐values database is also a valuable reference database (Gregory et al., 2007), with the caveat that there can be significant genome size variation between individuals in a species. For lineages rife with polyploidy, it is important to determine the ploidy level, either based on chromosome squash or by measuring pollen, stomata, or spore size (e.g., Li et al., 2012, 2017).

In addition to genome size, heterozygosity is another important consideration. When assembling short‐read shotgun sequences, heterozygous regions complicate graph structure and make it difficult to phase haplotypes. One way to reduce heterozygosity is to create inbred lines or doubled haploids, but this is time‐consuming and, furthermore, not every plant can be selfed for several generations or passed through anther culture and regenerated. For gymnosperms, inbreeding for just one generation might take decades, but certain species have large mega‐gametophytes that might provide enough haploid DNA for sequencing. Some ferns, on the other hand, are capable of intra‐gametophytic selfing (Haufler et al., 2016), which creates a complete homozygous sporophyte in one generation and can be effectively treated as a haploid in genome sequencing.

### *K*‐mer frequency distribution

**Figure 2:**
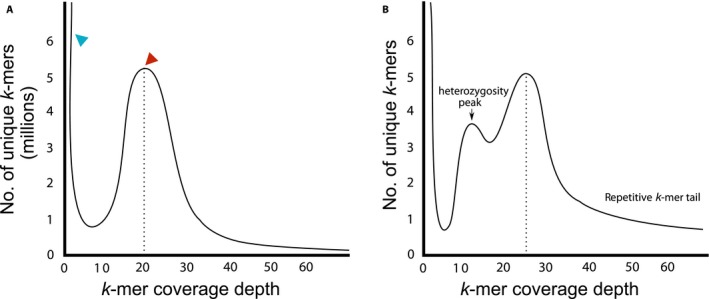
Hypothetical *k*‐mer frequency plots, showing a low‐heterozygosity genome (A) and a highly heterozygous and repetitive genome (B).

To discern if the chosen individual is appropriate for genome sequencing, a powerful and simple approach is to use raw Illumina DNA shotgun reads (Illumina, San Diego, California, USA) to infer genome size, repeat percentage, and heterozygosity. A *k*‐mer distribution refers to all the possible subsequences of length *k *that are contained within a string (or a set of strings) of nucleotides, such as a genome assembly or collection of sequencing reads. Figure 2A shows a hypothetical *k*‐mer frequency plot (*K* = 31) from shotgun Illumina sequencing data. The *x*‐axis shows the number of times a given *k*‐mer (e.g., ATGCTAGCTAACTAGACTACTAAGCTAGCAT) appears in the Illumina reads, and the *y*‐axis shows the number of unique *k*‐mers at that frequency. For example, the red arrow marks where 5 million unique *k*‐mers are found exactly 20 times. The first peak close to 1 (blue arrow) is largely due to sequencing errors that created abundant unique *k*‐mers of low frequency. The second peak (red arrow) represents the sequencing coverage, meaning that the majority of *k*‐mers were sequenced 20 times. The genome size can then be approximated by the total number of *k*‐mers (the area under the curve), divided by sequencing coverage.

A *k*‐mer frequency plot can also be used to estimate the level of heterozygosity in an individual. *K*‐mers from a heterozygous site will have half of the sequencing coverage compared to the homozygous region, creating an intermediate peak or shoulder halfway to the genome coverage peak (Fig. 2B). The higher this peak (or shoulder), the higher the sample heterozygosity. Similarly, *k*‐mers from repetitive regions will have a much higher representation than the mean coverage, and show up as a high frequency shoulder toward the right of the frequency plot (Fig. 2B). *K*‐mer plots can be generated by the Jellyfish package (Marçais and Kingsford, 2011) or KmerGenie (Chikhi and Medvedev, 2014), from where a few lines of R script can then be used to estimate the genome size (for tutorials see http://bioinformatics.uconn.edu/genome-size-estimation-tutorial/). It should be noted that the sequencing depth has to be sufficiently high, at least 30× coverage or more, in order for peaks to emerge.

The percentage and content of repetitive elements in a genome can also be inferred by shotgun Illumina DNA sequencing data before assembly. Sequence reads that cover as little as 1% of the genome size can be used as input to programs like RepeatExplorer (available as a free Galaxy server at http://www.repeatexplorer.org/; Novák et al., 2013) or Transposome (Staton and Burke, 2015). Random, low‐coverage shotgun reads are clustered by nucleotide similarity and overlap, then annotated against a plant repeat database like Repbase (http://www.girinst.org/repbase/; Bao et al., 2015) or one derived from a closely related genome.

Given the relatively low cost of Illumina sequencing and its usefulness in several ways before and after genome assembly, generating Illumina shotgun DNA sequence data is a critical step when starting a genome assembly project.

### DNA quality and quantity

The quantity, purity, and integrity of DNA often dictate the quality of the final genome assembly and therefore should not be ignored. For long‐read sequencing with Pacific Biosciences (or “PacBio”) sequencing systems (Pacific Biosciences, Menlo Park, California, USA), over 10 μg of DNA with 30–50‐kbp average fragment size is usually needed to obtain optimal results with the current chemistry. To obtain the best DNA possible, one could use the classic nuclei preparation protocols, although grams of starting plant materials are necessary (e.g., Zhang et al., 1995). QIAGEN MagAttract (QIAGEN, Valencia, California, USA) and Bionano IrysPrep (Bionano Genomics, San Diego, California, USA) are kit‐based alternatives. The integrity of DNA can be roughly and quickly visualized by conventional agarose electrophoresis, but to obtain a more accurate estimation of DNA fragment size, pulse‐field electrophoresis or the Agilent TapeStation (Agilent Genomics, Santa Clara, California, USA) should be used. The DNA purity should also be high, as contamination of salts and proteins will likely inhibit enzyme activity during library preparation. The NanoDrop (Thermo Fisher Scientific, Waltham, Massachusetts, USA) or other spectrophotometers that can measure 260/280 and 260/230 nm absorbance readings should be used for assessing sample purity, as DNA, proteins, and salts absorb at 260 nm, 280 nm, and 230 nm, respectively. A general rule of thumb is that 260/280 values of purified DNA should fall between 1.8–2.0, whereas 260/230 should fall between 2.0–2.2. Although spectrophotometry is useful to determine the purity of DNA, it is not appropriate for quantifying total genomic DNA for sequencing or for purified sequencing libraries. Qubit Broad Range (Thermo Fisher Scientific) or other double‐stranded DNA fluorescence‐based quantification is the preferred DNA quantification method for total genomic DNA.

## SEQUENCING PLATFORMS

There are two major types of sequencers, one that generates short reads (<300 bp) massively, accurately, and cheaply (e.g., Illumina and BGISEQ‐500 [BGI, Shenzhen, China]), and a second type that produces longer reads (>10 kbp) but inaccurately (7–12% error rate) and with a much lower throughput (e.g., PacBio and Oxford Nanopore [Oxford Nanopore Technologies, Oxford, United Kingdom]). For de novo sequencing, the PacBio or Nanopore long‐read technology is preferred and can be realistically done for a small‐ to moderate‐sized genome (<1 Gbp). Generating sufficient long‐read coverage for larger genomes might be prohibitively expensive, and a hybrid approach—combining both short and long reads—would be more feasible. For a more in‐depth review on sequencing technologies, see Goodwin et al. (2016).

### Illumina

The Illumina platforms have been the main workhorse for genome sequencing; they are able to create massive sequencing data cheaply and with a low error rate. Currently one lane of Illumina HiSeq4000 costs approximately US$2500, and outputs 90 to 100 Gbp of paired‐end 150‐nucleotide reads. For a genome of 1 Gbp, this throughput translates into impressive 100× coverage. The downside, however, is that the read length is short: 150 nucleotide maximum for HiSeq and 300 nucleotide for MiSeq. In addition, the library insert size usually cannot go beyond 800 nucleotides, providing only short‐range information. Therefore, for de novo sequencing, additional scaffolding approach is needed to achieve a reasonably good assembly.

### BGISEQ

BGI, a prominent sequencing provider and biotech company in Shenzhen, China, recently unveiled its own sequencer, BGISEQ‐500. This sequencer is based on Complete Genomics' nanoball technology for creating sequencing clusters, which is different from the bridge amplification method used in Illumina. The specification of BGISEQ‐500 is reportedly comparable to Illumina HiSeq2500, with similar read length, throughput, and error rate (Goodwin et al., 2016; Mak et al., 2017; Huang et al., 2017). This platform has not yet been widely adopted, however, and the pros and cons of BGISEQ‐500 over Illumina are still to be determined.

### PacBio

While the platforms discussed above only generate short‐read information, single‐molecule sequencing such as PacBio and Oxford Nanopore can read long DNA molecules (>10 kbp) without prior amplification. It is thus extremely useful for de novo sequencing of plant genomes. The disadvantage, however, is that the current single‐molecule sequencing technologies all have a high error rate—ranging from 10–15%—and come with a higher price tag per base than Illumina. One PacBio Sequel SMRT cell (2.0 chemistry and 10‐h movie) costs roughly US$1250 and yields 5 Gbp of long‐read data (>10–20 kbp). Given that at least 40× coverage is recommended for PacBio‐only assembly, a 1‐Gbp genome will cost US$10,000. It should be stressed that DNA quality, particularly the fragment length, matters tremendously for PacBio (and also for Nanopore) sequencing.

### Oxford Nanopore MinION

MinION is similar to PacBio in terms of read‐length, throughput, and error rate, but the entire sequencer is packaged into a USB‐sized device that is highly portable. The portability stands out among all the sequencers—all it requires is a moderately modern laptop and the library preparation can be completed in as little as 10 min. Because of this, MinION has been used in the International Space Station, as well as in arctic and other remote research stations. The genome of *Solanum pennellii* Correll (1–1.1 Gbp) was recently assembled based entirely on Nanopore data (Schmidt et al., 2017), and Michael et al. (2017) reported that an *Arabidopsis thaliana* genome (~135 Mbp) could be de novo sequenced by just one MinION flow cell. It is therefore possible to DNA barcode, genotype, or even sequence entire plant genomes real‐time in the field. The era of “mobile genomics” might soon be coming, although the hurdle now is how to efficiently extract high‐quality DNA outside of laboratories.

Oxford Nanopore is rapidly evolving both in terms of scalability and library preparation methods. For instance, the available GridION system is able to concurrently run up to five of the MinION‐sized flow cells with integrated computing power. Flow cell pricing is currently as low as US$300 each when purchased alongside the capital cost of the machine. Each of these five GridION flow cells are regularly able to generate more than 5 Gbp of long‐read data, with throughput varying with different DNA input quality, size selection, and library types. Library preparations for DNA vary from 5‐min transposase‐based rapid kits (longer reads, but lower throughput) to more traditional ligation‐based preparations (shorter reads, but maximum throughput). Reads can also be generated from a single strand of DNA (1D sequencing) or from a newly released library preparation that consecutively sequences both strands of a complementary DNA molecule (1D^2^ sequencing) for improved accuracy.

## GENOME ASSEMBLY

Assembling a genome is like solving a jigsaw puzzle, but an extremely difficult one. There are two assembly approaches, based either on de Bruijn graph (DBG) or Overlap‐Layout‐Consensus (OLC). The OLC assembly method first finds the overlaps among all the sequencing reads, from where a string graph is created to lay out the contigs. OLC then takes all the reads constituting each contig to create a consensus sequence. PacBio or Nanopore long reads are best assembled by the OLC assemblers, such as Canu (Koren et al., 2017), FALCON (Chin et al., 2016), and miniasm (Li, 2016). Notably, by taking advantage of long reads, FALCON‐Unzip (Chin et al., 2016) can potentially phase and assemble individual haplotypes and would be particularly useful for highly heterozygous genomes. OLC, on the other hand, is not designed for short reads, as overlaps between short sequences could be incorrect and it is computationally impossible to calculate pairwise overlaps among billions of reads. DBG is better suited to deal with massive short‐read data. DBG takes a counterintuitive approach to solve the genome assembly problem, by first shredding the already short reads into even shorter *k*‐mers. The rationale is that the connections among *k*‐mers can be much more easily constructed, and the resulting de Bruijn graph can be traversed to derive the contigs. Numerous DBG‐based assemblers have been developed, such as SOAPdenovo (Luo et al., 2012), ALLPATH‐LG (MacCallum et al., 2009), Velvet (Zerbino and Birney, 2008), ABySS (Jackman et al., 2017), and Platanus (Kajitani et al., 2014).

Getting high enough PacBio or Nanopore coverage for a large genome is not always possible, but one can reduce the cost by generating cheap short‐read data and adopting a hybrid assembly approach. This can be done in MaSuRCA (Zimin et al., 2017b), which first extends short reads into “super‐reads,” and uses these super‐reads to turn long reads into “mega‐reads.” These processed reads can then be assembled by OLC. Several giga base–sized plant genomes have been assembled in this way (Zimin et al., 2017b, 2017c).

## GENOME SCAFFOLDING APPROACHES

To improve genome assembly, it is critical to obtain long‐range information to orient and order contigs into scaffolds. Traditionally, this has been done either by generating a physical map or by constructing and sequencing bacterial artificial chromosome (BAC) libraries, both of which are laborious and costly. Fortunately, there are a few clever library preparation methods and new technological advances that make genome scaffolding much more cost‐effective and feasible for small labs.

### Mate‐pair library

This library preparation method takes long DNA fragments (>1 kbp) and self‐ligates them into circles, bringing the distant ends together. The circular DNAs are then cut, and the short fragments containing the joined junction are selected. From these fragments, the two distant parts of the genome can then be sequenced by standard Illumina paired‐end sequencing. Mate‐pair libraries, however, are not easy to construct, requiring a large quantity of high‐molecular‐weight DNA, and usually have low complexity (i.e., with many duplicates), thereby wasting the sequencing output. Given that PacBio and Nanopore read lengths regularly exceed 10–30 kbp in length, Illumina mate‐pair libraries are quickly falling out of favor.

### Hi‐C

In eukaryotic cells, nuclear DNA wraps around histones and is packed into a complex three‐dimensional chromatin conformation. Within this structure, two DNA strings packed in close proximity might be coming from two distant regions of chromatin, and such spatial relationships can be leveraged to create long‐range sequence information (Liu and Weigel, 2015). The Hi‐C method first cross‐links DNA to histones in vivo to preserve the chromatin conformation. DNA is then digested, allowing spatially close, but physically distant, DNA fragments to be ligated. The resulting libraries can be sequenced on the Illumina platform. Hi‐C has been shown to be able to create chromosomal‐level genome assemblies—given a draft assembly and the expected chromosome number, the Hi‐C scaffolder uses a statistical model to piece contigs together into individual chromosomes. In addition, native plant chromosomes are unlikely to be cross‐linked with foreign DNA, and contaminations can therefore be identified and excluded. The most notable feature of Hi‐C is that it does not require high‐molecular‐weight DNA (the cross‐linking, digestion, and ligation are done in vivo), thus providing a solution to materials that resist DNA extraction. Dovetail Genomics (Santa Cruz, California, USA) and Phase Genomics (Seattle, Washington, USA) offer commercial solutions to Hi‐C sequencing and scaffolding, often at a total cost between US$10,000 and US$20,000. Dovetail Genomics also has a Hi‐C variant, called the Chicago library; instead of cross‐linking in vivo, this method applies exogenous histones to pure DNA extraction to recreate chromatin structure in vitro but produces shorter inserts than in vivo Hi‐C. Recently, in vitro and in vivo Hi‐C have been used to improve numerous plant genomes, including lettuce (Reyes‐Chin‐Wo et al., 2017), barley (Mascher et al., 2017), amaranth (Lightfoot et al., 2017), and quinoa (Jarvis et al., 2017).

### BioNano optical mapping

The goal of optical mapping is to create a genomic restriction map, thereby providing a backbone to scaffold the contigs (Chaney et al., 2016). It starts with ultra‐high‐molecular‐weight DNA (>150 kbp) and uses single‐stranded restriction endonucleases to create nicks at specific recognition sites. Fluorescent nucleotides are then incorporated at each of the nicked sites. To visualize the labeling pattern, the DNA is applied onto a chip, where each molecule enters a nanochannel and becomes linearized. The intervals between fluorescent labels can be imaged precisely and in a high‐throughput fashion, thus enabling the construction of a genome‐wide restriction map. This restriction map can be paired with an in silico digestion of the genome assembly, allowing for scaffolding and assembly correction. Notably, multiple enzymes can be used to produce separate but complementary optical maps. The “two enzyme” approach mitigates a previous limitation of the technology, where nearby restriction sites on opposite strands would lead to DNA molecule breakage. Several high‐quality plant genomes have recently been scaffolded and improved with optical mapping, including maize (Jiao et al., 2017), garden asparagus (Harkess et al., 2017), and *Oropetium* Trin. (VanBuren et al., 2015), and the technique is even feasible for individually flow‐sorted chromosomes (Staňková et al., 2016).

### 10× Genomics

At the heart of 10× Genomics is their Chromium microfluidic controller. This platform, which resembles a toaster, enables massive partitioning of the input genomic DNA into oil droplets (a dozen or so DNA molecules per droplet), within which library preparation is performed and, importantly, each partition receives a unique barcode. Because reads with the same barcode can only come from one of the few DNA molecules, the long‐range information can be bioinformatically deduced to produce “linked” reads. In addition, homologous (or homeologous) DNA molecules are unlikely to be included in the same droplet, so that each haplotype will receive its own barcode and can be distinguished. For a highly heterozygous or polyploid individual, 10× Genomics linked reads can potentially provide a phased genome. Like many other scaffolding approaches, the length of DNA molecules determines the quality of long‐range information. The advantages of 10× Genomics technology, however, are that it only requires 1 ng of input DNA and is a more economical way to obtain long‐range information than Hi‐C or optical mapping (one 10× library costs roughly US$1400 to construct).

### Next steps: Assembly validation and annotation

The next necessary step to producing a genome usable by a larger community is to annotate it for genes, repetitive elements, and other regulatory and non‐coding regions. Annotation can first be used as an objective measure to judge the completeness of the assembly. BUSCO (Simão et al., 2015) is a useful tool to annotate an assembly to identify genes that are typically present in single‐copy across major lineages. A genome assembly that recovers full‐length copies of most of these typically single‐copy BUSCO genes would suggest that the sequencing and assembly approach successfully captured a significant amount of the expected gene content. After validating the completeness of the assembly, genome‐wide annotation tools tailored to plants, such as MAKER‐P (Campbell et al., 2014), can automatically perform and integrate gene and repeat annotations from several tools.

## DISCUSSION

What is then the best way to sequence a plant genome of 500 Mbp, 1 Gbp, or 5 Gbp? Choosing between the various sequencing and scaffolding approaches could be overwhelming. Paajanen et al. (2017) recently applied almost all of the methods mentioned above (with the exception of Nanopore) to assemble the *Solanum verrucosum* Schltdl. genome, and this could be used as a good benchmark reference. The general strategy is to generate long reads (by PacBio or Nanopore) if possible, which will give far better assembly than one based entirely on short reads. The recommended long‐read coverage is at least 40–50×; for a 500‐Mbp, 1‐Gbp, and 5‐Gbp genome, this roughly translates to US$5000, US$10,000, and US$50,000, respectively. A hybrid approach, using both short and long reads, can potentially be used for larger genomes. Generating 100× Illumina and 20× PacBio coverage for a 5‐Gbp genome would be approximately US$35,000; this is significantly less expensive than the PacBio‐only approach, but the assembly quality might be lower. If funding is limited but high‐molecular‐weight DNA is attainable, the 10× Genomics library with Illumina sequencing could yield a genome comparable to that from PacBio or Nanopore, but with a lower cost. In this case, a 500‐Mbp genome would cost roughly US$4000 (US$1400 for library generation and US$2600 for sequencing) and a 1‐Gbp genome would cost approximately US$5800 (US$1400 for library generation and US$4400 for sequencing).

The draft genomes usually have thousands of contigs; depending on the purpose, further scaffolding might not be necessary. For example, the gene space could be considerably well captured from such draft genomes, and can be readily used to infer species phylogeny or to examine gene family evolution. Draft genomes, on the other hand, may not be suitable for synteny analyses and examining genome structural evolution. Hi‐C and optical mapping might potentially bring the draft genomes to chromosomal‐level assembly, although that would require an additional US$10,000–20,000.

Currently, a few international consortia are aiming to broadly sequence plant genomes across the phylogeny. The 10,000 Plants Genome Sequencing Project, or 10KP, is planning to sequence over 10,000 plant and algae species, and will be done at BGI with the BGISEQ‐500 platform (Cheng et al., 2018). Another effort, the Open Green Genomes Initiative (OGG), is funded by the Joint Genome Institute and focuses on a few dozen phylogenetically important plants to generate high‐quality reference genomes (https://jgi.doe.gov/csp-2018-leebens-mack-open-green-genomes-initiative/). Unlike 10KP, OGG will incorporate PacBio as the sequencing platform. It is important to note that both of these initiatives follow the open data philosophy; therefore, we recommend that you check with 10KP and OGG before embarking on your own sequencing adventure. They may already have what you need!
